# Photo-Induced Room-Temperature Gas Sensing with a-IGZO Based Thin-Film Transistors Fabricated on Flexible Plastic Foil

**DOI:** 10.3390/s18020358

**Published:** 2018-01-26

**Authors:** Stefan Knobelspies, Benedikt Bierer, Alwin Daus, Alain Takabayashi, Giovanni Antonio Salvatore, Giuseppe Cantarella, Alvaro Ortiz Perez, Jürgen Wöllenstein, Stefan Palzer, Gerhard Tröster

**Affiliations:** 1 Electronics Laboratory, Swiss Federal Institute of Technology (ETH) Zürich, Gloriastrasse 35, 8092 Zürich, Switzerland; dausa@ife.ee.ethz.ch (A.D.); talain@student.ethz.ch (A.T.); giovanni.salvatore@ife.ee.ethz.ch (G.A.S.); giuseppe.cantarella@ife.ee.ethz.ch (G.C.); troester@ife.ee.ethz.ch (G.T.); 2 Laboratory for Gas Sensors, Department of Microsystems Engineering (IMTEK), University of Freiburg, Freiburg, Germany; benedikt.bierer@imtek.uni-freiburg.de (B.B.); alvaro.ortiz.perez@imtek.de (A.O.P.); juergen.woellenstein@imtek.uni-freiburg.de (J.W.); 3 Fraunhofer Institute for Physical Measurement Techniques, Freiburg, Germany; 4 Department of Computer Science, Universidad Autónoma de Madrid, Francisco Tomás y Valiente 11, 28049 Madrid, Spain; stefan.palzer@uam.es

**Keywords:** a-IGZO, gas sensor, flexible electronics, thin-film transistor, NO_2_

## Abstract

We present a gas sensitive thin-film transistor (TFT) based on an amorphous Indium–Gallium–Zinc–Oxide (a-IGZO) semiconductor as the sensing layer, which is fabricated on a free-standing flexible polyimide foil. The photo-induced sensor response to NO_2_ gas at room temperature and the cross-sensitivity to humidity are investigated. We combine the advantages of a transistor based sensor with flexible electronics technology to demonstrate the first flexible a-IGZO based gas sensitive TFT. Since flexible plastic substrates prohibit the use of high operating temperatures, the charge generation is promoted with the help of UV-light absorption, which ultimately triggers the reversible chemical reaction with the trace gas. Furthermore, the device fabrication process flow can be directly implemented in standard TFT technology, allowing for the parallel integration of the sensor and analog or logical circuits.

## 1. Introduction

Health complications arising from urban air pollution are among the top ten causes of death in high-income countries [1]. Most pollutants originate from fuel combustion in either mobile sources, such as combustion-powered vehicles, or stationary sources, like power plants [1,2,3]. Several studies have demonstrated that, among the pollutants, nitrogen dioxide (NO_2_) directly affects human health [4] by damaging DNA, or causing asthma [5] and other chronic pulmonary diseases [6]. The consequences of urban NO_2_ exposure contributed to an estimated 75,000 premature deaths throughout the European continent in 2012 [2]. Although the NO_2_ emission limits in leading markets have been progressively tightened, recent disclosures e.g., the Volkswagen diesel scandal, showed that the emissions of over half of on-road light-duty diesel vehicles exceed these certification limits [2,7].

As a direct result, the demand and interest in devices for health or environmental monitoring, such as smart wearable systems, have been growing rapidly. In this field, flexible electronics, e.g., sensors for UV-light [8], temperature [9] or gas [10,11] are emerging. One particularly appealing application is chemical gas sensing, which may be achieved using sensors based on metal oxide (MOX) semiconductors. Due to their low cost and high robustness, they are among the top candidates as a functional layer [12] and several groups have demonstrated the use of MOX semiconductors for NO_2_ sensing [13,14].

State-of-the-art MOX based gas sensors are used in resistive readout mode and usually require operation temperatures between 200 °C and 400 °C. Crystalline Tin dioxide (SnO_2_), Zinc oxide (ZnO) and Titanium dioxide (TiO_2_) are currently the most prominent materials, which account for about 70% of the overall MOX gas sensor applications [15]. Nowadays, one major research focus is the implementation of these materials into nano-sized structures, such as nanowires, nanobelts, nanorods or nanoparticles to increase the surface-to-volume ratio and therefore to increase the sensitivity [16]. Nevertheless, to achieve room-temperature operation and integration into wearable devices, new materials and different sensor structures need to be exploited. One alternative approach for chemical gas sensing with MOX semiconductors is the implementation into field effect transistors (FETs). The FET based gas sensor was presented in 1975, using a catalytically active gate material [17]. The observation that a shift in the electrical characteristics occurs due to polarization phenomena in the metal-semiconductor interface has led to a large number of different device types, usually produced in standard silicon technology [18]. Subsequent developments directly used the FET channel material as gas sensitive layer. The interaction between analyte and semiconductor leads to an electron transfer between them that changes the carrier concentration, mobility and semiconductor work function and ultimately results in a response of the transistor current [19]. Here, we utilize the recent developments in flexible thin-film transistor (TFT) technology [20] to fabricate a chemical sensor, whose channel material also acts as the gas sensitive layer. The integration of this gas sensor into a wearable device requires two key elements: mechanical flexibility and compatible fabrication processes of sensor and circuitry. Since flexible plastic substrates usually prohibit the use of high operating temperatures, we investigate the applicability of amorphous Indium–Gallium–Zinc–Oxide (a-IGZO) based TFTs as room temperature-operating gas sensors. In the field of flexible electronics, a-IGZO has received much attention due to its high electron mobility and compatibility with low temperature deposition processes [21]. Nevertheless, there exists just a few reports on a-IGZO gas sensors [22,23,24,25,26] that are based on a rigid substrate and resistive readout. As the interaction of the semiconductor with the trace gas relies on the availability and number of free charge carriers at the surface, UV-light can be used to generate electron-hole pairs, instead of traditional heating [23,24,27]. The gas sensor presented here shows sensitivity to NO_2_ in the ppm region with negligible hysteresis behavior. We perform a material analysis of the a-IGZO layer using X-ray diffraction (XRD), ultraviolet-visible (UV-Vis) spectroscopy, scanning electron microscopy (SEM) and atomic force microscopy (AFM), and relate the sensor behavior to its physical mechanisms. Finally, the cross-sensitivity to humidity is evaluated. The presented sensor with a compatible fabrication process to circuitry is paving the way for the implementation of a-IGZO based gas sensors in wearable applications.

## 2. Experimental and Results

### 2.1. Fabrication

**Figure 1:**
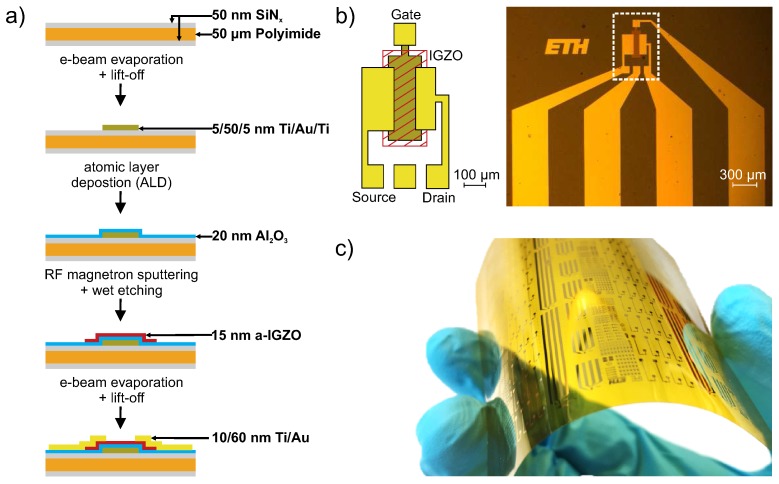
(**a**) schematic fabrication flow of the gas sensitive thin-film transistor (TFT) including the respective layer thicknesses. (**b**) layout of the TFT indicating the different layers and dimensions (**left**) and a macroscopic picture of the fabricated device (**right**). The TFT is highlighted with dashed lines. (**c**) photograph of the fabricated sensors on the flexible polyimide substrate.

The devices are fabricated on free-standing 50 μm thick polyimide foil (Kapton E, DuPont, DA, USA) in a bottom gate inverted staggered configuration. The fabrication flow and the schematic device cross-section is displayed in Figure 1a. First, the substrate is cleaned in acetone and 2-propanol for 5 min each with the help of sonication. Subsequently, a 24 h bake out at 200 °C is performed to remove residual solvents. Afterwards, a 50 nm thick layer of SiN_x_ is deposited on both sides of the substrate by plasma-enhanced chemical vapor deposition (PECVD) at 150 °C to inhibit further degassing and to promote the adhesion of the following layers. The gate metal consisting of Ti/Au/Ti (5/50/5 nm) is electron beam evaporated and patterned by photolitography and lift-off. Then, a 20 nm thick Al_2_O_3_ insulator layer is grown by atomic layer deposition (ALD) at 150 °C followed by a 15 nm thick a-IGZO layer deposited by room temperature RF magnetron sputtering using an InGaZnO_4_ target. The semiconductor layer is structured into islands and gate vias are formed in the insulator both by wet chemical etching. Finally, the drain/source (Ti/Au 10/60 nm) contacts are deposited by electron beam evaporation and structured by photolitography and lift-off. Similarly to what presented for TFTs, readout systems (composed by analog and digital circuits) can also be realized with the same thin-film technology. Figure 1b shows a sketch of the layout and a micrograph of a device after fabrication. A photograph of the fully fabricated flexible sample is presented in Figure 1c.

### 2.2. Physical Properties and Sensing Mechanism

The properties of the a-IGZO semiconductor layer are analyzed by UV-Vis absorption spectroscopy, an XRD measurement, SEM imaging and AFM.

**Figure 2:**
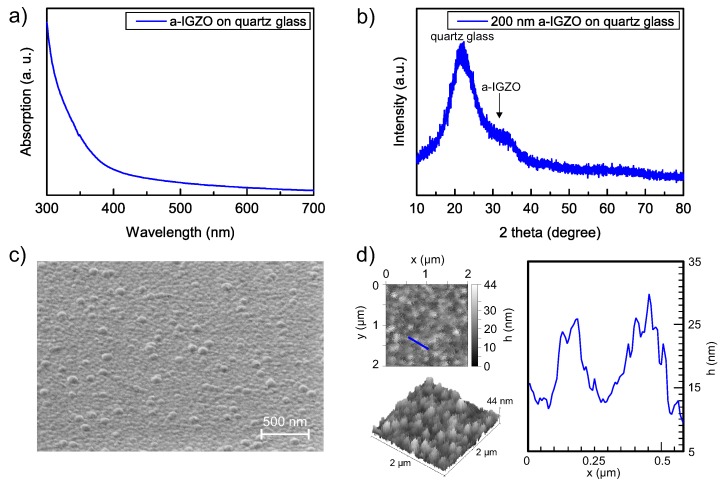
(**a**) ultraviolet-visible (UV-Vis) absorption spectra of 15 nm thick a-IGZO and (**b**) X-ray diffraction (XRD) of a 200 nm thick a-IGZO layer, both deposited on quartz glass. (**c**) scanning electron microscopy (SEM) image of the a-IGZO semiconductor layer; (**d**) atomic force microscopy (AFM) of the a-IGZO surface including the 2D and 3D height profile and a representative line profile. SEM and AFM imaging are performed on the fabricated TFT gas sensor.

Figure 2a shows the optical absorption spectrum of 15 nm thick a-IGZO. The layer is deposited on a quartz glass plate to avoid signal losses due to a highly absorbing polyimide substrate layer. The absorption value increases for light wavelengths smaller than the bandgap of 3.05 eV, which correlates to 406 nm [8]. To verify the amorphous structure of the RF-sputtered thin-film, an XRD measurement is presented in Figure 2b. Here, a 200 nm thick a-IGZO layer on quartz glass is used to increase the a-IGZO signal intensity and to obtain a well-known background signal. The first broad peak around 2 theta = 22 degrees corresponds to the quartz glass substrate [28] and the plateau between 30 and 35 degrees indicates a-IGZO [29].

The surface topology of the semiconductor layer is studied using SEM and AFM imaging and is shown in Figure 2c,d, respectively. Both measurements are performed on the fabricated TFT gas sensors. As reported in our previous work, the semiconductor forms a homogeneous layer that conforms to the underlying material stack (i.e., polyimide, SiN_x_, Ti/Au/Ti, Al_2_O_3_ stack) and the visible grains are related to the wavy surface of the SiN_x_ layer [30]. The average surface roughness determined from the AFM measurement is 4.727 nm, thus increasing the surface to volume ratio, which is preferable for the sensor operation. Due to the absorption spectrum of the a-IGZO, a UV-LED with a peak wavelength of 375 nm is used to generate free charges in the semiconductor [23,31], which will ultimately trigger the reversible chemical reaction with the trace gas. 

**Figure 3:**
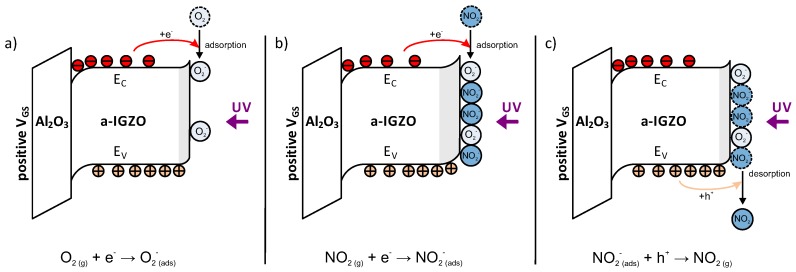
Schematic band diagram of the TFT sensing layer and gate insulator under UV-illumination and in contact with gas. Under illumination with UV-light, the generated electron-holes pairs are separated to the conduction and valence band, respectively. This results in an increased number of charge carriers thereby an increased transistor drain current (I_D_). (**a**) oxygen molecules from the atmosphere adsorb at the semiconductor surface acting as surface-acceptors and therefore forming a back-channel depletion as indicated in the band diagram. Due to this depletion region, the transistor I_D_ decreases. (**b**) As soon as the NO_2_ comes in contact with the sensor, it also adsorbs at the surface acting as an electron-acceptor. The back channel depletion area will increase and the transistor I_D_ decreases further. (**c**) After returning to normal atmosphere without NO_2_, the adsorbed NO_2_^−^ molecules desorb from the surface by the help of a positive charge from the semiconductor valence band. The back-channel depletion width and the I_D_ return to the states described in (**a**).

First, the photo-generated electron-hole pairs are separated to the conduction and valence band, respectively. This increase in charge carriers leads to an increased transistor drain current I_D_. As soon as the a-IGZO is in contact with the atmosphere, as shown in Figure 3a, the oxygen adsorbs at the semiconductor surface (Equation (1)), acting as an acceptor molecule and forming a back-channel depletion area. Due to the partial depletion, the I_D_ decreases accordingly [32]:

(1) $$O_{2}+e^{-}\to {{O_{2}}^{-}}_{\left(\mathrm{ads}\right)}.$$

When NO_2_ enters the atmosphere, it also adsorbs at the semiconductor surface as presented in Figure 3b. By taking one negative charge from the conduction band, the NO_2_ chemisorbs as NO_2_^−^ (see Equation (2)) [33]. The electron affinity of NO_2_ is significantly higher compared to the one of O_2_ [34]. The back-channel depletion width will increase, which results in a decreased I_D_. The change in I_D_ correlates directly with the NO_2_ concentration, since the atmospheric O_2_ concentration can be considered constant:

(2) $${\mathrm{NO}}_{2}+e^{-}\to {{{\mathrm{NO}}_{2}}^{-}}_{\left(\mathrm{ads}\right)}.$$

Since a-IGZO is an n-type semiconductor, the reactions with O_2_ and NO_2_ will also occur in a attenuated form without UV-illumination due to free electrons in the conduction band. These surface mechanisms occur due to the homogeneous non-porous a-IGZO layer, as described in the conduction model for MOX gas sensors in [35]. A-IGZO based TFTs are known to be influenced by humidity [36]. Nevertheless, as already proposed for other metal oxide semiconductors [37] and shown in Figure 3c, photo-generated holes can recombine with the previously trapped electrons of NO_2_ promoting the desorption of the gas molecules (Equation (3)):

(3) $${{{\mathrm{NO}}_{2}}^{-}}_{\left(\mathrm{ads}\right)}+{h^{+}}_{\left(\mathrm{photo}-\mathrm{generated}\right)}\to {\mathrm{NO}}_{2(g)}.$$

When H_2_O molecules adsorb at the metal-oxide surface, the free carrier concentration at the back-channel increases, which counteracts the depletion because charge is transferred between water molecules and the semiconductor surface (Equation (4)) [38]:

(4) $${H_{2}O}_{\left(g\right)}\to {{H_{2}O}^{+}}_{\left(\mathrm{ads}\right)}+e^{-}.$$

The interactions between semiconductor surface and gas molecules and the formation of a partially depleted channel are reported for zinc tin oxide TFTs and H_2_O [39], which is in agreement with the proposed mechanisms.

### 2.3. TFT Characterization and Gas Measurements

**Figure 4:**
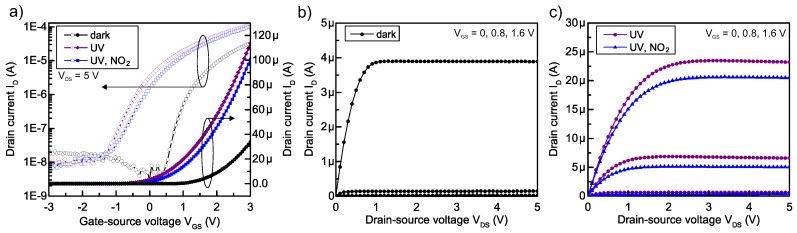
Electrical characteristics of the a-IGZO TFT (W/L = 280 μm/80 μm) gas sensor in dark condition, under UV-light illumination and while exposed to UV-light and 5 ppm NO_2_. For all measurements, the background oxygen was set to 20%. The measurement under UV-light was performed after 2 min illumination. (**a**) transfer characteristics and (**b**,**c**) output characteristics.

The TFT sensor response is measured with a two channel source-measure unit (SMU, Agilent B2902A, CA, USA). Figure 4 shows the transfer (I_D_-V_GS_) and output characteristics (I_D_-V_DS_) of a TFT gas sensor under dark condition (initial status), under UV-light illumination and while exposed to UV-light and 5 ppm NO_2_ gas.

In the dark condition, the TFT has a threshold voltage of V_Th_ = 0.87 V and an effective saturation mobility of μ_sat_ = 13.34 cm^2^V^–1^s^–1^. The sensor operating point is chosen in the saturation regime at a drain-source voltage V_DS_ of 5 V and a gate-source voltage V_GS_ of 0.8 V. Due to the incident UV-light, the V_Th_ and therefore the transfer characteristic are negatively shifted, as shown in Figure 4a and reported in [8]. In the presence of NO_2_, the transfer characteristic is shifted in a positive direction, resulting in a decreased I_D_, as also shown in the output curves of Figure 4c. The observations in the electrical characteristics are in agreement with our proposed sensing mechanism.

**Figure 5:**
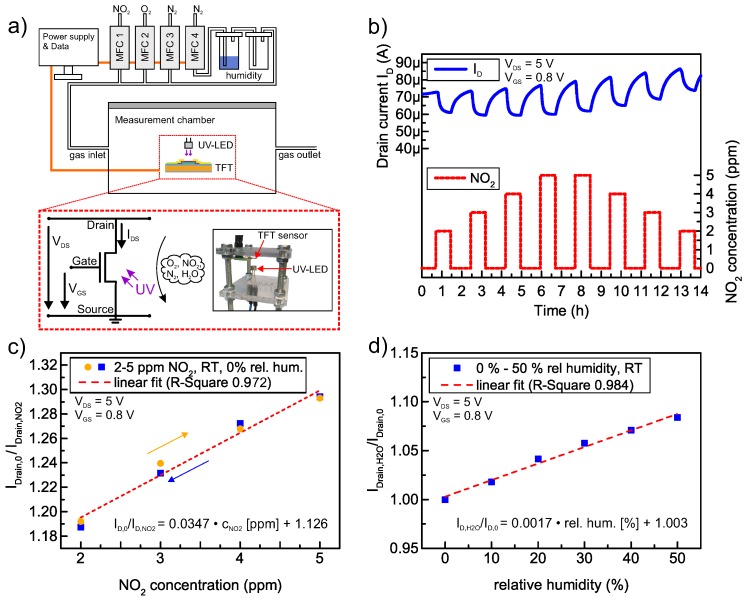
(**a**) schematic of the measurement setup. The desired gas mixture is prepared by four mass flow controllers (MFC 1-4), each connected to a different gas species. The relative humidity is changed by the ratio between dry (MFC 3) and wet (MFC 4) nitrogen. The TFT gas sensor, as well as the UV-light source are placed inside the measurement chamber (see photograph). The biasing and measurement scheme is presented in the inset; (**b**) I_D_ raw signal to 2–5 ppm NO_2_ with 20% background oxygen, 0% relative humidity at room temperature; (**c**) normalized sensor signal to 2–5 ppm NO_2_. The arrows indicate the sweep direction of the NO_2_ concentration; (**d**) cross-sensitivity to humidity (20% oxygen, RT) as normalized sensor signal.

The TFT sensor response is characterized in a custom-built laboratory apparatus, which allows for a automated simulation of various trace gas compounds and concentrations, including the relative humidity and background oxygen [40]. The time needed for a complete gas exchange inside the measurement chamber is estimated as 1.8 min, which is neglected in further calculations and fitting procedures. The measurement setup and biasing scheme are schematically presented in Figure 5a. The I_D_ of the TFT is used as the gas dependent sensor signal and the UV-intensity is set to 17 mW·cm^–2^. For all measurements, the O_2_ concentration is set to 20%, which resembles atmospheric conditions. The raw I_D_ signal at 2–5 ppm NO_2_ at 20% oxygen, 0% relative humidity and room temperature is shown in Figure 5b. Compared to the TFT characteristic in Figure 4, the I_D_ is significantly increased due to continuous UV-exposures over long periods of time and, as explained in Figure 3, the current decreases as soon as the sensor is in contact with the trace gas. We observe a drift in the sensor baseline that can be attributed to photo-induced charge trapping at oxygen vacancies within the a-IGZO or at the a-IGZO/Al_2_O_3_ interface as reported in our previous work [8]. Nevertheless, this effect does not influence the reaction of analyte gas with the semiconductor surface. To analyze the dependence between gas concentration and sensor signal, the I_D_ response is fitted with the exponential function $I\left(t\right)=A\;\cdot \;exp^{\left(-t/\psi \right)}+c$. The fits show an average R^2^-value of 0.9973 ± 0.0013. The extracted time constants τ for adsorption and desorption of NO_2_ are $\tau_{adsorption}=13.5\pm 3.6$ min and $\tau_{desorption}=50.2\pm 2.9$ min. Figure 5c presents the normalized response of the fitted steady state values, which are sufficiently reached after 100 minutes by extrapolating the respective functions I(t) to t = 100 min. The response to NO_2_ is defined by $I_{Drain,0}/I_{Drain,NO_{2}}$. In the analyzed gas concentrations, we observe an almost hysteresis free change of I_D_ in the range of 19% to 29%. Even though one expects a logarithmic behavior, linear fitting can be applied in the analyzed NO_2_ range to estimate the sensitivity, resulting in a value of 3.47%/ppm. It is worth mentioning that the sensor response is quite slow, but, through exponential fitting, the gas concentration can already be estimated before reaching the steady state values. The response time of the sensor could be improved by inducing heat, e.g., by implementing a buried microheater structure, since the surface response of metal oxide based gas sensors to NO_2_ exposure is directly affected by temperature as shown for Cu(II)O in [14]. Furthermore, we evaluated the cross-sensitivity to humidity (defined by $I_{Drain,H_{2}O}/I_{Drain,0}$.) in the range of 0% to 50%. The device shows an almost linear dependence (R-square = 0.984) between relative humidity and the normalized I_D_, as presented in Figure 5d. By linear fitting, as indicated by the dashed line, the cross-sensitivity yields a value of 0.17%/rel.hum.(%).

## 3. Conclusions

We presented, for the first time, a photo-induced a-IGZO based gas sensitive TFT that operates at room temperature. The fabrication flow on a flexible free standing polyimide foil is compatible with state-of-the-art flexible TFT technology to allow the parallel integration of the gas sensor and readout circuits. The physical mechanisms, i.e., the photo-generation of free charge carriers and the resulting reactions between semiconductor surface and oxygen, NO_2_ and humidity are explained by means of a band diagram and chemical reaction equations and the principle is confirmed by experiments. The sensor shows an almost hysteresis free response of 3.47%/ppm to NO_2_ in the investigated range of 2–5 ppm with 20% background oxygen at room temperature. Furthermore, a nearly linear cross-sensitivity to humidity is observed between 0% and 50% relative humidity, which is small compared to the NO_2_ response. Previous studies showed that our TFT technology allows a stable operation of the TFTs while bent to a tensile radius down to 6 mm without significant changes in the electrical characteristics and the response to UV-light [8]. Thus, future research should be focused on the gas sensing performance under mechanical bending. Further aspects that have to be considered are the influences of small temperature variations within the room temperature range and the correction of the baseline-drift.
